# Mental health related service contacts in children with ‘sub-threshold/sub-clinical’ psychopathology in the Mental Health of Children and Young People in England national survey

**DOI:** 10.1192/j.eurpsy.2023.999

**Published:** 2023-07-19

**Authors:** T. Newlove-Delgado, F. Mathews, T. Ford

**Affiliations:** 1 Medical School, University of Exeter, Exeter; 2 Department of Psychiatry, University of Cambridge, Cambridge, United Kingdom

## Abstract

**Introduction:**

Population surveys often present prevalence estimates of children meeting criteria for psychiatric disorders, which are used to plan services. However, studies have shown that those with ‘subthreshold’ or’ subclinical’ symptoms also experience poorer outcomes, and benefit from identification and support.

**Objectives:**

This study uses data from the 2017 NHS Digital Mental Health of Children and Young People in England survey (MHCYP-2017), a large probability sample, to examine prevalence of ‘sub-threshold’ difficulties and contact with services.

**Methods:**

Secondary analysis of data from MHCYP-2017, using data on 6,718 children aged 5 to 16. The main measures of mental health were the Strengths and Difficulties Questionnaire (SDQ), a validated dimensional measure, and the Development and Wellbeing Assessment (DAWBA), a standardised diagnostic assessment which was clinically-rated to assign diagnoses based on ICD-10 and DSM-V criteria. Parents also reported on mental health related service contacts for their child in the previous year. Descriptive analysis reported the proportion of participants with ‘sub-threshold’ difficulties. This was defined as a high or very high score on the parent-rated SDQ total difficulties score and/or impact score, but not meeting criteria for a DSM-V diagnosis on the DAWBA. Levels of service contact in this group were reported.

**Results:**

According to provisional findings (subject to final weighting strategy), 7.2% (95% CI 6.5-7.8%, n=486) of 5- to 16-year-olds fell into this ‘sub-threshold’ category, 79.1% (95% CI 78.1-80.1%, n=5,295) had no disorder and did not have raised impact or total difficulty scores on the SDQ, and 13.7% (95% CI 12.9, 14.6%, n=937) had a DSM-V diagnosis. Almost half of those with ‘sub-threshold’ difficulties had contact with professional services in the previous year (47.4%, 95% CI 42.8, 52.1%). Teachers were the most commonly reported professional service contact (39.8%, 95% CI 35.3, 44.4%). Contact with child mental health specialist services was reported in 6.5% (95% CI 4.5, 9.1%).

**Conclusions:**

This initial analysis demonstrates that a small but significant (on a population level) proportion of children in this sample had elevated levels of mental health difficulty and/or impact on functioning but did not meet criteria for a disorder. As these data are cross-sectional, it is possible that some children may meet, or have met, diagnostic criteria at an earlier or later point. Almost half of this group had had mental health related contact with a teacher, providing opportunities for early intervention, but only a small proportion had contact with specialist services. These analyses can inform planning and targeting of support for children who may not meet criteria for specialist services.

**Disclosure of Interest:**

None Declared

